# Guild of Friends of the Infirm in Mind

**Published:** 1887-08-13

**Authors:** H. Hawkins

**Affiliations:** Chaplain, Colney Hatch


### Guild of Friends of the Infirm in Mind.

By the Rev. H. Hawkins, Chaplain, Colney Hatch.

"What can man's wisdom do,
In the restoring his bereaved sense?"—*King Lear.*

For some years a little Society has been in existence, called the "Association of Friends of the Infirm in Mind." Its object is to befriend a few at least of the friendless poor who are either inmates of lunatic asylums or have been recently discharged convalescent. There is a large field for work. It is reckoned that about 70,000 pauper patients* are inmates of asylums and similar institutions. These are composed of individuals belonging to almost all professions, trades, handicrafts, miscellaneous employments—to nearly every nation and religion. Infants, little children, young men and women, the middle-aged, the elderly, and the very old, are found among these legions. The majority are women. In one asylum alone there are, besides men, more than 1,300 women, married, widows, and single, governesses, shopwomen, domestic servants, artificers of various kinds, etc. Of course, many of these are more or less friendless, separated from all acquaintances by distance, estrangement, death, or other causes. It is some of these friendless ones that the Association endeavours to help and comfort. Moreover, an experienced mental physician has recently asserted that "it is impossible to deny that there is reason to fear some real increase of recurring insanity."† Out of so large a number suffering or just recovering from some sad form of insanity, it may readily be supposed that not a few are, as regards the outside world, entirely forsaken and forlorn. The object of this little Association is to comfort and help some of these. Although officially unconnected with any particular institution, practically it has worked on behalf of some of the patients in the Colney Hatch Asylum, near London. Some brief particulars of its quiet work may not be uninteresting. First, to speak of visits paid to friendless patients. It may be an elderly, forlorn woman who has known brighter days, or some poor creature whose past misconduct has brought her within asylum walls, or a young girl neglected by or far off from her friends; or perhaps a foreigner, a desolate "stranger in a strange land," or some other unvisited patient who sorely needs a friend's sympathy and attention, the kind offices, that is, of some one from without, some friend of *her* own, not rendered as *official* duty, but "all for love, and nothing for reward." But how is this boon to be secured? How can a lonely inmate of an asylum yearning for friendship have her craving gratified? Let us suppose the chaplain or some other official of the institution to be acquainted with some lady living near who would be disposed to visit any selected patient. The name of a suitable case is mentioned to her. On a visiting day, and within visiting hours (regulations must be scrupulously observed), she calls at the asylum, mentions the patient's name, is shown into the visiting-room, and there has a first interview with her poor sister. As some intimation would have been given to the patient of the intended visit, it would not be quite unexpected. A few kindly words, and a little tact on the part of the visitor, may be sufficient to initiate a pleasant acquaintanceship. A considerate visitor will

* Number of patients in county asylums, workhouses, etc., at beginning of 1886, 71,663.

† *Insanity, and its Prevention,* p. 136. By D. D. Hack Tuke. 1878.

not go empty-handed. Some little gift, especially if suited to the tastes of the recipient, will render the visit more welcome; so the kind friend takes with her that sure passport to the female heart, a little tea and sugar, or it may be a little cake, or fruit, a few sweetmeats; or again, a small coin, or illustrated paper, or amusing book, or indeed any trifle likely to afford pleasure, and not forbidden by rules. In some cases a good understanding might be established by the offer of a packet of snuff or "screw" of tobacco. A Christmas or birthday card, or, at the beginning of the year, an almanack, would give no little pleasure.

After an interview of moderate length the visitor takes her leave, having first engaged to renew the acquaintance at no distant date. Visits need not be repeated frequently. Once a quarter would be sufficient to sustain the patient's interest, without being burdensome to the visitor. The benefit might be extended if *two* patients were seen, either together or at alternate visits.

It is within the writer's knowledge that acquaintanceship thus formed between an otherwise unvisited patient and a benevolent neighbour, has been sustained, with mutual gratification, for years, and might be kept up after convalescence and departure. And a patient on her dying bed has spoken gratefully of the kindness shown by a lady visitor. Nor is the benefit on the side of the patient alone. The visitor is profited also by the acquisition of a humble but devoted friend; by enlarged sympathies; and, perhaps, by a sense of thankfulness for the gift of a sound mind.

But, in some cases, persons might be unable, on account of distance or other reason, to pay *personal* visits, and yet might be desirous to contribute something towards the alleviation of the trials of asylum life.

When a visit cannot be made in *person* it may through the *post*. A newspaper or magazine (if illustrated, all the better), addressed to some inmate, or to the care of an official, may relieve the wearisomeness of an hour. An old *Graphic*, or *Illustrated*, the *British Workman*, or *Gospeller*, the *Family Herald*, are examples of what would be sure to be acceptable. Many communications reach Colney Hatch from persons who have never visited it, or seen any of its inmates. From Cornwall, Devonshire, Kent, Essex, Jersey, Hants, Herts, Suffolk, Somerset, Wilts, Middlesex, Sussex, the post or rail have brought tokens of goodwill from friends personally unknown. A considerate friend, unknown by sight, once brought, all the way from America, some tasteful gifts for distribution among the patients. At Christmas time the considerately planned "Hospital Pillow Mission" has cheered many poor folk who have never seen the givers. If any benevolent persons disposed to confer pleasure, through the post, will confer with the Chaplain of Colney Hatch, he will gladly suggest some practical way of carrying out their kind intentions. An almanack for the New Year, a Christmas, Easter, or birthday card would be, in their seasons, welcome mementoes. Flowers by parcels post have often cheered patients. Perhaps nothing is, as a rule, more acceptable than a few stamps, or a small postal order representing the "wherewithal" to buy some trifling luxury next time a visit is paid to some shop by the patient herself, or some one on her behalf.

One very useful way in which benefit might be conferred on asylums would be the recommendation for service therein, as attendants, of young men and women of good character, physically and morally well qualified for dealing with the infirm in mind, and with some special aptitude for the work; for asylum work requires the most efficient workmen and workwomen it can secure. If ladies would sometimes direct the attention of such young persons as they would value in *their own* households to the calling of nurses in asylums, they would suggest a vocation which might prove advantageous both to themselves and the community in which they ministered. The wages are liberal; special encouragements are given, and there are opportunities of promotion.

There is so large a field for work, both among patients within asylums and friendless convalescents recently discharged, that it might even justify the organisation of a religious society (such as a sisterhood) devoted to this special branch of Christian work. Abroad, there does exist an Order, working chiefly, if not exclusively, on behalf of the infirm in mind.* But no such *Anglican* Order (as far as is known) exists. Might it not be practicable to found an Order within our own communion, which (adapting itself to the arrangements made for the care of the mentally afflicted in this country) should specially endeavour to promote the interests of the infirm in mind? 1. The work would have the attraction of novelty. No such Order exists among us. The pioneers of this movement would break fresh ground. 2. There would be a wide and useful field of labour among the many thousands of patients of this class residing in public asylums and elsewhere.

A Sisterhood existing to promote the well-being of the insane would itself offer regular intercessions on their behalf, and would also engage the prayers of others; it would pay personal visits to any friendless patients in asylums or elsewhere, who would be likely to benefit by such attention and sympathy; and it would reach others, to whom personal visits might not be practicable, through the medium of the post, and in this manner would comfort and cheer—by kindly messages or little considerate gifts—many to whom such acts of sympathy and attention might prove very consolatory, or even restorative. Within the scope, moreover, of such a Religious Order would come the provision of "Homes" for the temporary reception of friendless convalescents, and any after-care which their various circumstances might require. And it would not be their least service if they inaugurated some scheme for educating persons as attendants on the insane, and imparting to them a higher tone and view of their work than generally prevail at present. One shilling only is payable on membership, and there is no further subscription or payment.

Any person interested in the above subject, and desirous of obtaining more information about it, is invited to communicate with the Rev. H. Hawkins, care of the Editor, THE HOSPITAL, who will gladly reply to any letter he may receive.

* Bruges, with branch at Burgess Hall, Sussex.

